# Supplementary material for: Using full-text content to characterize and identify best seller books: A study of early 20th-century literature
Source: PLoS One. 2024 Apr 26;19(4):e0302070. doi: 10.1371/journal.pone.0302070 (PMC11051604; doi:10.1371/journal.pone.0302070)
Supplement: S1 File — (PDF) [file pone.0302070.s001.pdf]

Using Full-Text Content to Characterize and Identify Best Seller Books – Supporting Information

Giovana D. da Silva [1], Filipi N. Silva [2],* Henrique F. de Arruda [3],
Bárbara C. e Souza [1], Luciano da F. Costa [4], and Diego R. Amancio [1]
[1] *Institute of Mathematics and Computer Science – USP,*
Avenida Trabalhador São-carlense, no 400,
CEP 13566-590, São Carlos, SP, Brazil
[2] *Indiana University Network Science Institute,*
Bloomington, Indiana, 47408, USA
[3] *CENTAI Institute, Corso Inghilterra 3, 10138, Turin, Italy*
[4] *São Carlos Institute of Physics – USP,*
Avenida Trabalhador São-carlense, no 400,
CEP 13566-590, São Carlos, SP, Brazil

(Dated: April 11, 2024)

I. IDENTIFYING BEST SELLERS USING NON-FULL-TEXT CONTENT

To assess whether the partial content of the books would also be sufficient to discriminate best sellers from non-best sellers, we replicated the approach with the best-obtained result for $N = 1000$ and $N = 2000$ first words from each book. The higher accuracy method consisted in modeling the books with bag-of-words, normalizing the vectors, and then applying logistic regression and cross-validation (both leave one out and 10-fold) to evaluate the results. The reason to choose 1000 and 2000 as the number of words is to have no more than ten pages per book.

For $N = 1000$, we obtained an accuracy of 0.52 using leave one out and an accuracy of 0.54 ± 0.13 using 10-fold. Alternatively, for $N = 2000$, we got 0.58 for leave-one-out and 0.58 ± 0.07 for 10-fold. If juxtaposed to the original results, where both cross-validations reached an accuracy of 0.75, it is possible to verify the need for more information for better class discrimination. Nonetheless, we emphasize that identifying the threshold between partial and full content that offers a satisfactory degree of separation between classes constitutes a reasonable research problem to be tackled by future works.

II. WHY IS ULYSSES, BY JAMES JOYCE, IN THE NON-BEST SELLER CLASS?

Detailing the story of Leopold Bloom, a Dublin publicity agent who condenses Odysseus' journey into just one day, Ulysses, by James Joyce, is a parody of the Odyssey, written by Homer. When published in 1920, the book was considered too obscene, too obscure, and too sensational, making few dare to read it in the first third of the century[1]. For describing aspects of human physiology (considered unpublishable), it was even censored in places like the United States and the United Kingdom. For this reason, it is reasonable that the book did not enter the best seller list in its publication year – one of its characteristics being its late recognition.

Our study lacks part of the temporal aspect of a best seller. That said, it does not evaluate books published in year X that became hugely successful in year $X + 15$ (being $X + 15 > 1923$), for example. As previously described, the appraised best seller list considers solely the number of sales in physical stores in each year between 1895 and 1923 (to ensure public domain criteria). This fact can be a limitation of the dataset used, which leaves room for future work to analyze other datasets (which may even include online sales numbers) in addition to other techniques. On the other hand, it can be seen not as a limitation but as a question: *what is a best seller?* Is a book published in year X that sold ten copies a year through year $X + 10$ *more of a best seller* than a book that sold ninety copies in its year of release and then none? The definition of a best seller is very open to interpretation and may vary from person to person.

Nonetheless, it is worth mentioning that our pipeline classified Ulysses as a best seller, as exhibited in Figure 1. So, there is an indication that our approach is insensitive to the aforementioned temporal aspect of the successes. Future work may also explore this issue.

We made available on GitHub[2] an interactive version of the plot from Figure 1. In it, it is possible to explore

* filsilva@iu.edu

[1] Shepard, R. F., 1972, "U.S. Sales Of the Book At 880,000". *The New York Times*, Feb. 2, accessed 23 June 2023, https://www.nytimes.com/1972/02/02/archives/us-sales-of-the-book-at-880000.html.

[2] https://htmlpreview.github.io/?https://github.com/giovanadanieles/bestsellersAnalysis/blob/main/visualization/interactiveVisualization_BoW_LDA.html

the books' authors and titles besides our classification results (i.e., whether the book was classified as a success or a non-success by our pipeline).

III. ASSESSING READABILITY SCORES AND TEXT FEATURES

Complementarily to one of our methods, which employs superficial characteristics of words (namely frequency), we separately gauged more inherent characteristics of the text. The purpose was to experiment if such information would offer a better discriminative power for the two categories of books studied. In that sense, we considered some readability indices and statistics related to the number and grammatical class of words and the number and style of sentences. To obtain such values, we employed a ready-made library called readability[3], which supports four types of statistics:

1. Readability grades (namely: Kincaid, ARI, Coleman-Liau, Flesch Reading Ease, Gunning Fog index, LIX, SMOG index, RIX, and Dale Chall index);
2. Sentence information (composed of data such as the average number of words per sentence, the average number of sentences per paragraph, and the average number of syllables per word, among others);
3. Word usage (which informs the total number of auxiliary verbs, conjunctions, and pronouns, among others);
4. Sentence beginnings (which measures the number of sentences that starts with pronouns or prepositions, the number of interrogative sentences, among others).

The pre-processing done was the one recommended by the library: tokens (words or punctuation) separated by space, one sentence per line, no line breaks within sentences, and paragraphs separated by one empty line. So that there was no bias arising from the size of each text, metrics such as the number of conjunctions used and the number of sentences beginning with an article were normalized (in this case, by the total number of words and sentences of each book, respectively).

As there were many measures and results to be analyzed (the library has a total of 35), and as all the results obtained within each of the four classes (readability, sentence info, word usage, and sentence beginnings) were very close, we brought one result of each for visualization, as exhibited in Figure 2. Nonetheless, no measure offered a fruitful result concerning class separation, so there was no further investigation in our study involving these features.

IV. ASSESSING PRECISION, RECALL, AND F1-SCORE METRICS

Supplementarily to the accuracy analysis depicted in the main text, we also gathered information concerning *precision*, *recall*, and *f1-score* of classification methods. Tables I, II, and III expose such metrics, considering only the bag-of-words modeling of the PS+PR dataset. We select specifically this case (and only this case) since (i) it holds the best-achieved accuracy (namely, 0.75) and (ii) all the obtained values are very close — a reflection of the fact that accuracy is an appropriate metric for the explored scenario. As an example, regarding the 0.75 accuracy (obtained for the LR classifier, with standardized data, and with both LOO and 10-fold cross-validations), we have comparable precision, recall, and f1-score (between 0.73 and 0.75).

TABLE I. **Classification precision for different models and arrangements using bag-of-words modeling.** Results for configurations M or $\hat{M}$ and leave-one-out or k-fold cross-validation.

	M		$\hat{M}$	
	LOO	10-fold	LOO	10-fold
KNN	0.64	0.65 ± 0.21	0.58	0.60 ± 0.20
LR	0.65	0.68 ± 0.18	0.75	0.73 ± 0.19
NB	0.64	0.60 ± 0.16	0.64	0.60 ± 0.16
DT	0.64	0.64 ± 0.20	0.64	0.64 ± 0.20
RF	0.71	0.69 ± 0.18	0.71	0.70 ± 0.19
SVM	0.65	0.67 ± 0.16	0.72	0.70 ± 0.16

TABLE II. **Classification recall for different models and arrangements using bag-of-words modeling.** Results for configurations M or $\hat{M}$ and leave-one-out or k-fold cross-validation.

	M		$\hat{M}$	
	LOO	10-fold	LOO	10-fold
KNN	0.64	0.63 ± 0.16	0.57	0.61 ± 0.20
LR	0.62	0.66 ± 0.13	0.75	0.70 ± 0.18
NB	0.58	0.54 ± 0.15	0.58	0.54 ± 0.15
DT	0.69	0.60 ± 0.12	0.69	0.60 ± 0.12
RF	0.61	0.61 ± 0.13	0.61	0.62 ± 0.12
SVM	0.63	0.66 ± 0.19	0.73	0.68 ± 0.13

[3] van Cranenburgh, A. *readability*, 2019 (v0.3.1). GitHub repository: `https://github.com/andreasvc/readability`.

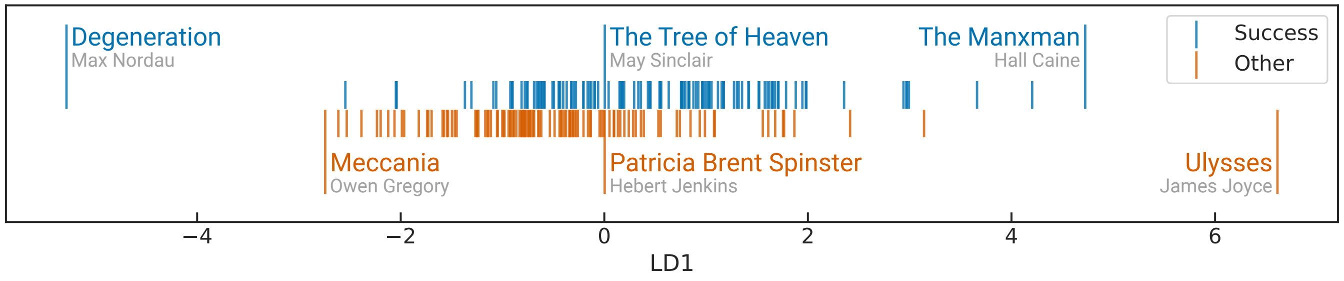

FIG. 1. Annotated LDA projection of bag-of-words representation of books. As one can see, Ulysses, by James Joyce, is in the same region as the titles predicted as successes by the proposed approach.

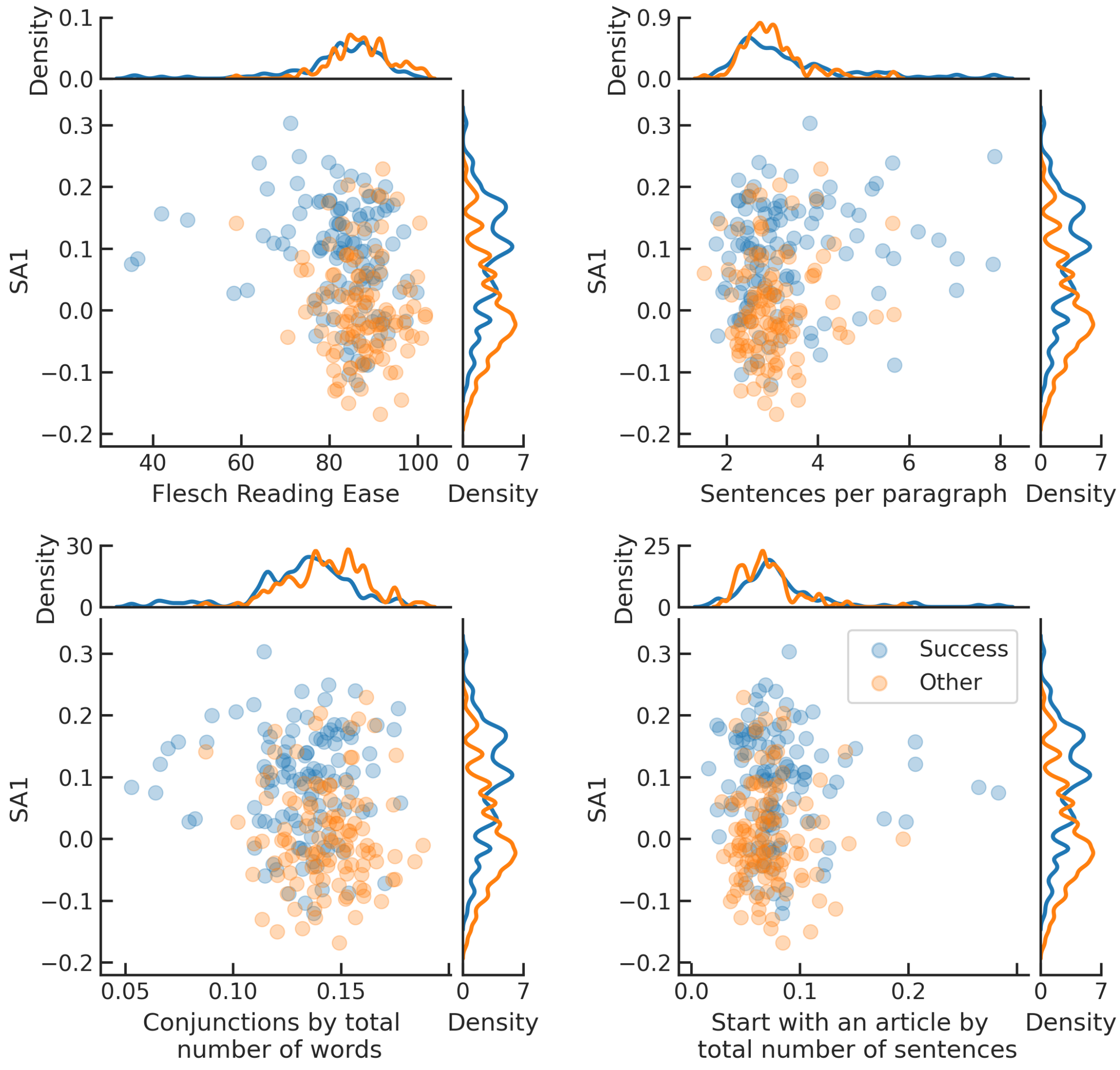

FIG. 2. On the x-axis, the new measures evaluated: Flesh Reading Ease, number of sentences per paragraph, number of conjunctions (normalized by the total number of words), and number of sentences starting with an article (normalized by the total number of sentences), respectively (reading order: left to right, top to bottom). On the y-axis, the formerly discussed SemAxis projection of the bag-of-words representation of the books. In blue are the successful instances of the dataset, and in orange are the non-successful ones. One can see that the new measures do not provide a degree of separability between classes.

TABLE III. **Classification f1-score for different models and arrangements using bag-of-words modeling.** Results for configurations M or $\hat{M}$ and leave-one-out or k-fold cross-validation.

	M		$\hat{M}$	
	LOO	10-fold	LOO	10-fold
KNN	0.64	0.62 ± 0.18	0.58	0.58 ± 0.18
LR	0.64	0.65 ± 0.12	0.75	0.70 ± 0.16
NB	0.61	0.56 ± 0.15	0.61	0.56 ± 0.15
DT	0.66	0.60 ± 0.13	0.66	0.60 ± 0.13
RF	0.66	0.63 ± 0.12	0.66	0.64 ± 0.12
SVM	0.64	0.64 ± 0.13	0.73	0.69 ± 0.13
