# Supplementary material for: Using full-text content to characterize and identify best seller books: A study of early 20th-century literature
Source: PLoS One. 2024 Apr 26;19(4):e0302070. doi: 10.1371/journal.pone.0302070 (PMC11051604; doi:10.1371/journal.pone.0302070)
Supplement: Attachment — Submitted filename: response.pdf [file pone.0302070.s003.pdf]

Dear Editor,

Please find attached a revised version of the manuscript "Using Full-Text Content to Characterize and Identify Best Seller Books", in which we have considered all the reviewers' comments. The most substantial changes made are marked in magenta in the revised version. Also attached is a list of responses to the reviewers' comments.

Yours sincerely,
The authors.

Reviewers' comments:

Reviewer #2:

The dataset used in this paper consists of 219 books published between 1895 and 1924. The title of this paper is "Using Full-Text Content to Characterize and Identify Best Seller Books". Therefore, one issue that needs to be addressed is whether the method in this paper is applicable to identification of current Best Seller Books. This is because the full-text content of current books can be accessed online. Therefore, the author needs to limit the title of the paper and conduct necessary discussions.

Answer: Thanks for pointing that out. We updated the paper title to *"Using full-text content to identify best sellers: a study of early 20th-century literature"*. In this way, we hope to reflect the period of the books analyzed, ensuring that the reader is aware of this information before starting to read. Furthermore, we added a paragraph in the *Conclusions* section that reaffirms that one of the limitations of this work is that we cannot analyze books published after the beginning of the 20th century due to copyright reasons. The paragraph is as follows:

> *(...) Concerning the limitations of the work, the two main points we stress are (i) the absence of modern books in the database and (ii) the absence of more modern modeling techniques. As previously discussed, the scarcity of books is due to copyright laws that protect the complete contents of modern books. Even though such content is found free of charge on the internet, we do not have the right to use it.*

Reviewer #3:

Pros:

1. The paper is organized and well written.

2. This paper shows a comparison analysis between the proposed model with numerous traditional classifiers.

3. The proposed method and results help publishing companies and writers.

Cons:

1. The results were not good (the average accuracy of 75%). These results may make the model's decisions untrusted.

Answer: Thanks for pointing that out. We recognize that 75% accuracy is not a lot, but we highlight the intricate nature of the problem, which makes it difficult to achieve high accuracies (such as 80 or 90%). To make this clear, we added an excerpt in the *Conclusions* section. The excerpt is as follows:

> *(...) In addition to these points, it is also worth mentioning that although an accuracy greater than 80 or 90% would be desirable, it would be unrealistic to predict the success of works with such a high outcome, given the intricate and multifaceted nature of such a task. Factors like marketing and trends can influence the popularity of books in ways difficult to predict or measure. Therefore, the 75% accuracy result becomes reasonable, although somewhat limited, if we think we are exploring solely the textual content of each book.*

2. It may be better to check deep learning like BERT or a large language model (LLM).

Answer: Thanks for pointing that out. In *Text embeddings* subsection we mention the following:

> *(...) More sophisticated techniques such as BERT and sentence BERT generate embeddings that capture richer context and semantic information of words or sentences. However, these techniques, similar to W2V and GloVe, are limited to a small number of tokens and can not be applied to large portions of texts, such as entire books.*

However, to highlight this limitation (which may not have become so clear during reading), we added a new excerpt in a *Conclusions* section paragraph that mentions the limitations of the work. The excerpt is as follows:

(...) Regarding modeling, more modern techniques, such as BERT, do not deal well with long texts. As the median size of our dataset is approximately 90,000 characters, it would not be appropriate to apply such a technique. Even if the modeling yielded highly accurate results, they would not be reliable.

3. The pictures are blurring.

Answer: Thanks for your comment. We double checked, and the images are in good quality. The PLOS ONE submission system automatically converts the images from the PDF to lower quality versions. To access the figures in higher resolution, click on “download figure” in the PDF.

Note: It may be better to remove “Then” in the text “Then, to obtain quantitative and more objective results, we employed various classifiers”

Answer: Indeed, thanks for spotting it. We updated it accordingly.
