# Supplementary material for: Using full-text content to characterize and identify best seller books: A study of early 20th-century literature
Source: PLoS One. 2024 Apr 26;19(4):e0302070. doi: 10.1371/journal.pone.0302070 (PMC11051604; doi:10.1371/journal.pone.0302070)
Supplement: Attachment — Submitted filename: best_sellers_response_to_reviewers.pdf [file pone.0302070.s004.pdf]

Dear Editor,

Please find attached a revised version of the manuscript "*Using full-text content to characterize and identify best seller books: a study of early 20th-century literature*", in which we have considered all the reviewers' comments. The most substantial changes made are marked in magenta in the revised version. Also attached is a list of responses to the reviewers' comments.

Yours sincerely,
The authors.

Reviewers' comments:

Reviewer #3:

Pros:

1. The paper is organized and well written.
2. This paper shows a comparison analysis between the proposed model with numerous traditional classifiers.
3. The proposed method and results help publishing companies and writers.

Cons:

1. The obtained results, with an average accuracy of 75%, fall short of expectations, potentially undermining the reliability of the model's decisions. Despite the author's assertion that the results were reasonable, they lie in the middle ground between randomness (50%) and high accuracy (<99%). Additionally, depending solely on the accuracy metric is insufficient. The paper should include other evaluation metrics, such as precision, recall, and F1-score, to provide a more comprehensive assessment of the classification models.

Answer: Thank you for your comment. The proposed model aims to tackle the very complex task of predicting the success of a book solely based on its content. While a 75% accuracy rate may seem lower in terms of reliability, for instance, for deployment in the industry, we never anticipated the model would achieve high values anyway. We acknowledge that several external factors, beyond just the content, significantly influence whether a book becomes a bestseller. This includes the author popularity, adaptations into other media forms (e.g., movies inspired by the book), and the political and social context at the time of publication. Such an accuracy value showcases that the content seems to be an important factor in determining the success of books. Future work may improve these numbers by employing more sophisticated methods. For delivering the prediction task, future models could also incorporate content, author information, and more context in

order to achieve better performance. We included such a suggestion in the conclusions.

Concerning the other evaluation metrics, we added a new section (Section IV - Assessing precision, recall, and f1-score metrics) in the supplementary material, where we expose the precision, recall, and f1-score for the modeling/configuration that achieved the best-obtained results. As stated there, these metrics are not considerably gainful, as they yield results too similar to the accuracy. However, we recognize the importance of exposing them so that the reader can understand that (i) accuracy is an appropriate metric for this case and (ii) all the other metrics sustain the value of the accuracy.

2. Exploring deep learning models like BERT or a large language model (LLM) could be more beneficial. The revised paper (R2) notes that "BERT does not deal well with long texts," referencing a study that utilized only the first 510 tokens of extensive text. However, this truncation limits classifiers' awareness of most the input text. There are alternative techniques to handle lengthy text when applying BERT, such as segmenting the text into chunks to fit the model's input size. Moreover, LLMs exhibit a capability to manage larger input sizes.

Answer: Indeed, there are alternative methodologies in the literature, such as aggregating vector representations or using larger token sizes. However, even these approaches are constrained by a relatively small token limit, extending up to 32,000 tokens (e.g., the Longformer [1] and TransformerXL [2]). Considering that a typical book may contain between 70,000 and 120,000 tokens, these models still fall short of covering entire texts. Moreover, the inherent computational memory requirements for processing with large models add another layer of complexity. Additionally, pre-trained large language models may not be ideally suited for our analysis due to potential biases in their training datasets. For instance, a preliminary examination of ChatGPT revealed its ability to detect whether a book was a bestseller based solely on its title, indicating possible data contamination. Given our lack of control over the training datasets of these large language models, their applicability in our study is not clear.

[1] Beltagy, Iz, Matthew E. Peters, and Arman Cohan. "Longformer: The long-document transformer." arXiv preprint arXiv:2004.05150 (2020).

[2] Dai, Z., Yang, Z., Yang, Y., Carbonell, J., Le, Q. V., & Salakhutdinov, R. (2019). Transformer-xl: Attentive language models beyond a fixed-length context. arXiv preprint arXiv:1901.02860

Although fine-tuning and exploring the performance of large language models for predicting the success of books is beyond the current scope of our research, we propose in the conclusions that future studies should assess the efficacy of LLMs for this specific task. Two significant challenges must be addressed: the limited token size, which might be mitigated through a strategy of piecewise summarization, and ensuring the training dataset used in the model does not influence its analysis. One potential solution could involve using only inputs from books published after the model training, which poses challenges due to restricted access to contemporary works. Alternatively, models could be carefully trained with datasets excluding references to the analyzed books.

3. The dataset limitation is apparent, consisting of only 219 examples.

Answer: Thanks for your comment, we really appreciate that. We acknowledge the limitation of our dataset and added a new excerpt on the Conclusions to reinforce this restriction. The excerpt is as follows:

> *(...) Concerning the limitations of the work, the three main points we stress are (i) the absence of modern books in the database, (ii) the absence of more modern modeling techniques,* ***and (iii) the limitation in dataset size imposed by the number of available best-selling books.*** *(...) We are restricted by the number of books listed as best sellers and also available in the public domain. As previously stated, we can not leverage books that don't have free content. Also, best-selling books are scarce per nature: if all books were best-selling pieces, this study would not even exist.*
